# Constructing a model of the factors related to the job wellbeing of preschool teachers in China: a grounded theory study

**DOI:** 10.3389/fpubh.2024.1497629

**Published:** 2025-01-15

**Authors:** Li-Ying Nong, Chen Liao, Jian-Hong Ye

**Affiliations:** ^1^Normal College, Hezhou University, Hezhou, China; ^2^Faculty of Education, Beijing Normal University, Beijing, China; ^3^National Institute of Vocational Education, Beijing Normal University, Beijing, China

**Keywords:** empowering leadership, Job Demands-Resources model, job wellbeing, leadership style, preschool teachers, work pathways

## Abstract

**Introduction:**

As educational management research evolves, leadership styles are increasingly recognized as crucial managerial skills. Among various leadership approaches, empowering leadership has been found to significantly enhance employee job performance and satisfaction. However, there is limited research exploring the relationship between empowering leadership and the job wellbeing of preschool teachers in the educational sector.

**Methods:**

This study examines the relationship between empowering leadership and preschool teachers' job wellbeing using the Job Demands-Resources (JD-R) model. Semi-structured interviews were conducted with 12 preschool teachers and 12 kindergarten leaders in China. The qualitative data collected were analyzed using grounded theory to identify themes and construct a systematic model.

**Results:**

The findings suggest that empowering leadership of kindergarten principals is positively related to preschool teachers' job wellbeing. This relationship is mediated through stress pathways, such as work pressure and burnout, as well as through enhanced organizational support and a positive work environment. Furthermore, empowering leadership influences teachers' personal motivation and work engagement, which in turn impacts their job wellbeing.

**Discussion:**

The study highlights the importance of empowering leadership in improving preschool teachers' wellbeing. By addressing stress factors, enhancing organizational support, and promoting personal motivation, empowering leadership can foster a more supportive and engaging work environment. These findings have practical implications for leadership practices in early childhood education settings.

## 1 Introduction

Preschool education is a critical foundation for children's future learning and development, shaping their cognitive, social, and emotional skills (1). Preschool teachers are at the heart of this foundational phase, playing a key role not only in fostering children's learning but also in nurturing their creativity, emotional growth, and overall wellbeing (2). Due to lower financial investment in kindergartens in China compared to other OECD countries, preschool teachers in China generally receive lower salaries. Moreover, preschool teachers face various challenges and difficulties in their daily work, including demanding working conditions such as routine educational and nurturing tasks, collaborative education with families, paperwork, using ICT in teaching, and inspections by higher authorities (3). These challenges can lead to a range of issues that jeopardize their physical and mental health and work status, such as job stress and high turnover rates, all of which may have a strong correlation with their ability to achieve high levels of job wellbeing.

Wellbeing is an important indicator in educational outcomes, as highlighted in global frameworks such as the OECD 2030 targets (4) and the United Nations' Sustainable Development Goals (5). Maintaining positive and stable wellbeing is especially vital for preschool teachers, given their significant role in early childhood education (6). From the perspective of positive psychology, wellbeing is often seen as a psychological process, and individuals should properly deal with negative and positive emotions when faced with challenges and changes (7). The wellbeing of preschool teachers directly affects the quality of preschool education and the development of young children (8). Compared to other education stages, preschool teachers face additional challenges, including not only educational tasks but also caregiving responsibilities, making their work demanding and often stressful (9). In China, recent policies aimed at improving early childhood education have shaped the work environment for preschool teachers in significant ways. The “Three-Year Action Plan for Preschool Education” launched by the Ministry of Education (2020) aimed to expand the availability of preschool education and improve educational quality. While this policy increased access to preschool services, it also intensified the workload for teachers due to an increase in the number of children enrolled without proportional increases in teacher numbers or resources. This mismatch has resulted in high student-teacher ratios, which places immense pressure on teachers, often leading to stress and burnout (10). These conditions may adversely affect the wellbeing of early childhood teachers (11).

Moreover, the “Opinions on Strengthening Preschool Teacher Workforce Development” issued by the State Council (2018) aimed to improve teacher training and professional development opportunities. However, issues such as low wages, insufficient social recognition, and limited professional growth opportunities remain pervasive, exacerbating teachers' dissatisfaction and negatively impacting their wellbeing (12). Despite these efforts to improve training, the lack of corresponding financial support and recognition means that teachers continue to experience high levels of stress and job dissatisfaction. These policies demonstrate that while there have been efforts to enhance early childhood education, the lack of sufficient resources, support, and recognition significantly affects preschool teachers' wellbeing. The demands on preschool teachers are rising, but the support mechanisms, such as adequate pay, reasonable workload, and professional development, have not kept pace (8). This discrepancy between policy goals and on-the-ground realities leads to increased stress, burnout, and a decline in overall job satisfaction among preschool teachers (13).

The Job Demands-Resources (JDR) model is widely used to explore the relationship between job characteristics and employee wellbeing (14). For preschool teachers, the demands of both educational and caregiving responsibilities make their work particularly challenging. Schaufeli (59) suggested that leadership can influence employee burnout and engagement through stress and motivational pathways, which is highly relevant to preschool teachers, given their high levels of job demands and relatively limited resources. Studies using the JD-R model have shown that empowering leadership, whereby leaders share power with employees, promotes autonomy, participation, and job significance, thereby enhancing job wellbeing (15, 16). Among these strategies, empowering leadership, defined as a process whereby leaders share power with employees through a series of specific leadership behaviors, is also seen as enabling employee autonomy, promoting participation in decision-making, enhancing job significance, and expressing confidence (17). In the context of preschool education, empowering leadership can help address the unique demands faced by preschool teachers, such as managing classroom dynamics, supporting children's developmental needs, and handling administrative tasks (18). Effective leadership that provides emotional support, autonomy, and decision-making opportunities can directly impact preschool teachers' wellbeing by reducing burnout and fostering a positive work environment (73).

However, current research primarily focuses on the general relationship between job characteristics, work environment, and employee wellbeing (70), lacking specific insights into how leadership styles affect preschool teachers' wellbeing and the unique challenges they face (19). Therefore, this study, using the JD-R model as the theoretical framework, analyzed the relationship between empowering leadership and preschool teachers' wellbeing of empowering leadership on preschool teachers' wellbeing. This study employed grounded theory to explore the following three questions:

Q1. What are the work conditions and wellbeing of preschool teachers working in kindergartens in China?
Q2. How does the wellbeing of preschool teachers manifest under empowering leadership?
Q3. What factors are related to the well-being of kindergarten teachers in China?

## 2 Literature review

### 2.1 Job wellbeing in the educational context

From the perspective of positive psychology, job wellbeing is an important research topic (20, 21). In the context of organizational work, job wellbeing refers to employees' positive emotional and cognitive evaluations of their work. Studies have found that work resources, such as social support, significantly impact teachers' job wellbeing. Specifically, research on preschool teachers' wellbeing has primarily been quantitative, highlighting the effects of emotional exhaustion and poor working conditions on wellbeing and turnover intentions (22, 23). Preschool teachers often face unique challenges, such as low wages, long working hours, burnout, and stress, which result in a high level of occupational stress. Increasing their psychological capital and social support has been shown to reduce work stress and positively influence their wellbeing (6). In childcare institutions, preschool teachers' work environment contributes significantly to stress and symptoms of depression. Research using the JD-R model has found that work resources have a significant correlation with preschool teachers' wellbeing, with positive correlations between a supportive work environment, work atmosphere, and overall wellbeing (24). Thus, research on preschool teachers' job wellbeing emphasizes the importance of social support, reducing stress, and enhancing work performance.

### 2.2 Empowering leadership

Empowering leadership is a management style that plays a critical role in reducing job demands and enhancing motivation (25). It is a process in which leaders share power with employees by granting them autonomy, involving them in decision-making, and fostering a sense of meaningful work (17, 26). For instance, empowered leadership has been found to alleviate work stress by emphasizing the meaningfulness of work, expressing confidence, and promoting autonomy, ultimately contributing to improved organizational outcomes (27). Furthermore, Empowering leadership has been demonstrated to have a substantial relationship with employee motivation, work satisfaction, and overall emotional wellbeing (28, 29). The dual nature of the role of the preschool teacher, which encompasses both educational and caretaking responsibilities, makes empowering leadership a particularly crucial aspect of management (71). In addition, empowering leadership is particularly pertinent in that it enables leaders to address the distinctive demands faced by teachers, affording them greater autonomy and decision-making authority (30). Empowering leadership has been shown to increase preschool teachers' sense of responsibility and ownership, while also contributing significantly to their job satisfaction and emotional wellbeing (17, 74). When preschool teachers perceive that their leaders trust them and provide them with the freedom to make decisions, they experience a greater sense of control over their work, which in turn leads to improved motivation and less stress (31). Furthermore, empowering leadership can foster a supportive organizational culture in which teachers feel appreciated and encouraged, thereby reinforcing their commitment to the organization and enhancing their job wellbeing (32). In the context of preschool education, empowering leadership is especially important given the demanding nature of teachers' dual roles in educating and caring for young children. Effective leadership that provides resources and autonomy can help address the unique demands faced by preschool teachers, reducing burnout and supporting a positive work environment.

### 2.3 Application of the JD-R model in education

The JD-R model is a widely used framework for exploring the relationship between job characteristics and employee wellbeing (14). Schaufeli (59) extended the model to include leadership as a factor related to employee wellbeing through stress and motivational pathways. Work stress can be regarded as job requirements which come from the organization's demands on the job, and are the negative physiological or psychological responses caused by the individual's response to the organization's job demands (33). Empowering leadership, as part of this model, has been shown to effectively reduce job demands and enhance work engagement, ultimately influencing organizational outcomes (25). While the JD-R model has provided theoretical support for understanding work dynamics, its application in the kindergarten educational environment has been limited. Preschool teachers face unique job demands, as they must simultaneously fulfill both educational and caregiving roles, creating specific work requirements and resource needs. Thus, this study used the JD-R model to focus specifically on the work environment of preschool teachers, exploring how empowering leadership affects their wellbeing by influencing work resources and stress conditions. In addition, in the kindergarten work environment, teachers not only need to interact with children on a daily basis, but also need to assume the dual roles of teaching and caring for young children, thus forming the unique work requirements and resource needs of kindergarten teachers. Therefore, based on the JD-R model, this study specifically focused on the working environment of preschool teachers, and explored how empowered leadership correlates with teachers' work wellbeing by influencing work resources and stress conditions.

## 3 Methodology

Grounded Theory, as a social science research method, is widely used in fields such as education and sociology to explain and understand human behavior and social phenomena (34). It emphasizes collecting and analyzing data through a series of coding and analysis procedures, using inductive and interpretive methods to form and construct theories, thereby helping researchers to explain and understand research phenomena more deeply (35). Given its effectiveness in theory construction, this study adopted a qualitative research method and used Grounded Theory for in-depth analysis to explore the factors related to Chinese kindergarten teachers' job wellbeing. This study aimed to identify and understand the challenges faced by kindergarten teachers in the current educational environment, and their specific effects on their job wellbeing, thereby constructing a model of factors and processes correlated with kindergarten teachers' job wellbeing.

### 3.1 Procedure and participants

Guest et al. (68) suggested that determining the sample size in qualitative interviews typically involves selecting a sufficiently small sample to ensure that individual cases are thoroughly analyzed and each participant's voice is represented. In qualitative research, sample size is often guided by the concept of theoretical saturation, which is the point at which no new themes or insights emerge from additional data collection (75). For interpretive phenomenological research, a sample generally consists of 8–12 participants, allowing for in-depth analysis of each case. However, grounded theory often requires a slightly larger sample size to ensure a comprehensive understanding of the phenomena under study and to reach theoretical saturation (76). In this study, we selected a sample of 24 participants-−12 kindergarten leaders and 12 kindergarten teachers—using purposive sampling (Tables 1, 2). This approach was adopted to gain diverse perspectives based on participants' gender, years of service, and type of kindergarten. The sample size was determined based on the need to reach theoretical saturation, which was monitored throughout the data collection process. Saturation was continuously monitored during the data collection phase, and it was determined that it had been reached when the final interviews revealed no new codes, categories, or themes. This means that, by the end of the data collection, all key aspects of the phenomenon were well-represented, and additional data did not contribute new insights. Theoretical saturation in this study was further verified by comparing emergent categories across different participant groups (i.e., leaders and teachers) to ensure that the themes were consistently observed in both groups. All participants agreed to take part in the study, and semi-structured interviews were conducted from June 2022 to January 2023. The interviews were based on the JDR model to understand participants' perspectives on the relationship between empowering leadership and the job wellbeing of kindergarten teachers.

**Table 1:** Information on kindergarten principal interviewees.

**Interviewees**	**Gender**	**Experience (in years)**	**Kindergarten type**	**Interviewees**	**Gender**	**Experience (in years)**	**Kindergarten type**
01	Female	12	Public	07	Male	4	Public
02	Female	17	Public	08	Female	8	Private
03	Female	6	Private	09	Female	5	Private
04	Female	14	Public	10	Female	7	Public
05	Female	11	Private	11	Female	8	Public
06	Female	3	Private	12	Female	6	Public

F, female; M, male.

**Table 2:** Early childhood teacher respondent information.

**Interviewees**	**Gender**	**Experience (in years)**	**Kindergarten type**	**Interviewees**	**Gender**	**Experience (in years)**	**Kindergarten type**
01	Female	2	Public	07	Female	6	Public
02	Female	7	Private	08	Female	8	Private
03	Female	4	Public	09	Female	9	Public
04	Female	11	Public	10	Female	7	Private
05	Female	4	Private	11	Female	8	Public
06	Female	3	Private	12	Female	3	Private

F, female; M, male.

### 3.2 Interview questions

To better understand the reasons for preschool teachers' job wellbeing, we conducted interviews with two groups: kindergarten principals and preschool teachers, to explore the factors influencing preschool teachers' job wellbeing. The interview outline was divided into six major themes based on the research questions, with a total of 12 questions for kindergarten principals (see Table 3) and 12 for preschool teachers (see Table 4). These themes included empowering leadership, work stress, job burnout, organizational support, work engagement, and job wellbeing, with the aim of analyzing their views on and the factors correlated with preschool teachers' job wellbeing. The initial interview questions were reviewed, discussed, and revised by three experts in the field of educational research. The review process involved deleting redundant questions, adjusting for clarity and focus, and ensuring alignment with the factors influencing preschool teachers' job wellbeing. After the experts' approval, the final interview outline was confirmed. Subsequently, researchers entered real interview settings, allowing for flexible adjustment of the content and sequence of the interviews (36). This approach aimed to deepen and enhance the understanding of the identified and defined research questions from the perspectives of kindergarten leaders and teachers (37).

**Table 3:** Kindergarten principal interview outline.

**No**.	**Questions**
1	What do you understand by empowering leadership? Can you share your experiences with empowering practices in your kindergarten work? What actions have you taken, and why did you choose to use empowerment?
2	To which employees do you tend to delegate authority, and what tasks do you empower them with? Under what circumstances do you usually decide to empower?
3	What are the main sources of stress in kindergarten work? What impact do these stresses have?
4	What measures do you think can be taken to handle these work pressures? What actions would you take?
5	How do you perceive job burnout? Have there been instances of burnout among preschool teachers at your kindergarten?
6	What impact do you think this job burnout has? How would you address it?
7	Have you provided support, care, or assistance to preschool teachers in your work? How exactly have you done this?
8	What measures do you think can be implemented to support preschool teachers?
9	How would you describe the work condition of preschool teachers in the kindergarten? Can you provide examples?
10	What factors do you think affect their level of engagement at work? Can you provide examples?
11	Do you think job wellbeing is important? Why is it important?
12	What factors do you believe influence their job wellbeing? Can you provide examples?

**Table 4:** Preschool teachers interview outline.

**No**.	**Questions**
1	How do you understand the concept of “empowering leadership”? Have you experienced being empowered in your work? In which aspects of your work is this empowerment reflected?
2	To what extent do you think empowerment helps your work? What are the impacts?
3	Do you feel the work pressure is high? What do you think causes you to feel stressed?
4	How much impact do you think this stress has on you? How do you alleviate it?
5	Have you ever felt job burnout in your work? What do you think are the reasons for feeling burnt out?
6	What impact do you think job burnout has had on you?
7	Do you feel supported, cared for, and helped by your kindergarten, leaders, and colleagues in your work? Can you provide specific examples?
8	What kind of support do you hope to receive from the kindergarten, leaders, or colleagues? Why?
9	How would you describe your current level of engagement in your work? For example?
10	What factors do you think motivate you? What factors affect your level of work engagement? For example?
11	Where is your sense of happiness usually reflected in your work? What stands out the most?
12	What factors affect your sense of happiness at work? For example?

### 3.3 Data analysis

Coding is a process of representing a portion of audio or visual data with words or phrases that are inductive, significant, and extract key information. Therefore, based on familiarity with the textual materials, the researchers arranged and coded the interview data according to the collection objects and timing. The data were then categorized and archived according to different types, where PU and PR denoted, respectively, public kindergartens and private kindergartens; different interviewees were coded as numbers 1–24; A1–A54 represent categories of open coding, while the codes for the interview outlines range from 01 to 12. The data were then analyzed using the Maxqda software to facilitate further data analysis. For specific details, please refer to the Data Categorization and Coding (Table 5).

**Table 5:** Data coding.

**Code number**		**Code**	**Description of meaning**
1	First code	PU, PR	Indicates different interviewee codes: public kindergarten (PU), private kindergarten (PR)
2	Second code	1 to 12	Indicates different interviewee codes, kindergarten principals (indicated by the letter L), preschool teachers (indicated by numbers T01-12)
3	Third code	A1-A54/A1-A74	A represents categories of open coding
4	Fourth to fifth code	01 to 12	Represents interview outline number.

## 4 Results

This study employed grounded theory to conduct semi-structured interviews with 12 kindergarten principals and 12 preschool teachers. Through a systematic analysis of the data, three primary themes emerged, corresponding to the work conditions and challenges faced by preschool teachers, the mutual relationship between empowering leadership and their wellbeing, and the factors relating to their overall job wellbeing (see Appendix 1 data coding). In response to RQ1: What are the work conditions and wellbeing of preschool teachers working in kindergartens in China? The results indicate that preschool teachers face a range of challenging work conditions that are closely linked to their job wellbeing. First, regarding the Work Conditions and Challenges of Preschool Teachers, the analysis revealed that preschool teachers encounter a range of challenging work conditions. Key issues include heavy workloads, demanding caregiving responsibilities, administrative paperwork, and frequent interactions with families. Despite these pressures, participants also recognized positive aspects of their roles, such as the fulfillment derived from supporting children's growth and the camaraderie with colleagues.

Second, in response to RQ2: How does the wellbeing of preschool teachers manifest under empowering leadership? The results indicate that empowering leadership behaviors—such as granting autonomy, fostering trust, and providing support—play a key role in enhancing preschool teachers' job wellbeing. Regarding the Mutual Relationship between Empowering Leadership and Wellbeing, the study found a reciprocal relationship between empowering leadership and preschool teachers' wellbeing. On one hand, empowering leadership fosters wellbeing by reducing teachers' stress, enhancing their job satisfaction, and increasing their motivation. However, excessive empowerment without adequate support can lead to increased stress and role ambiguity.

**Figure 1:**
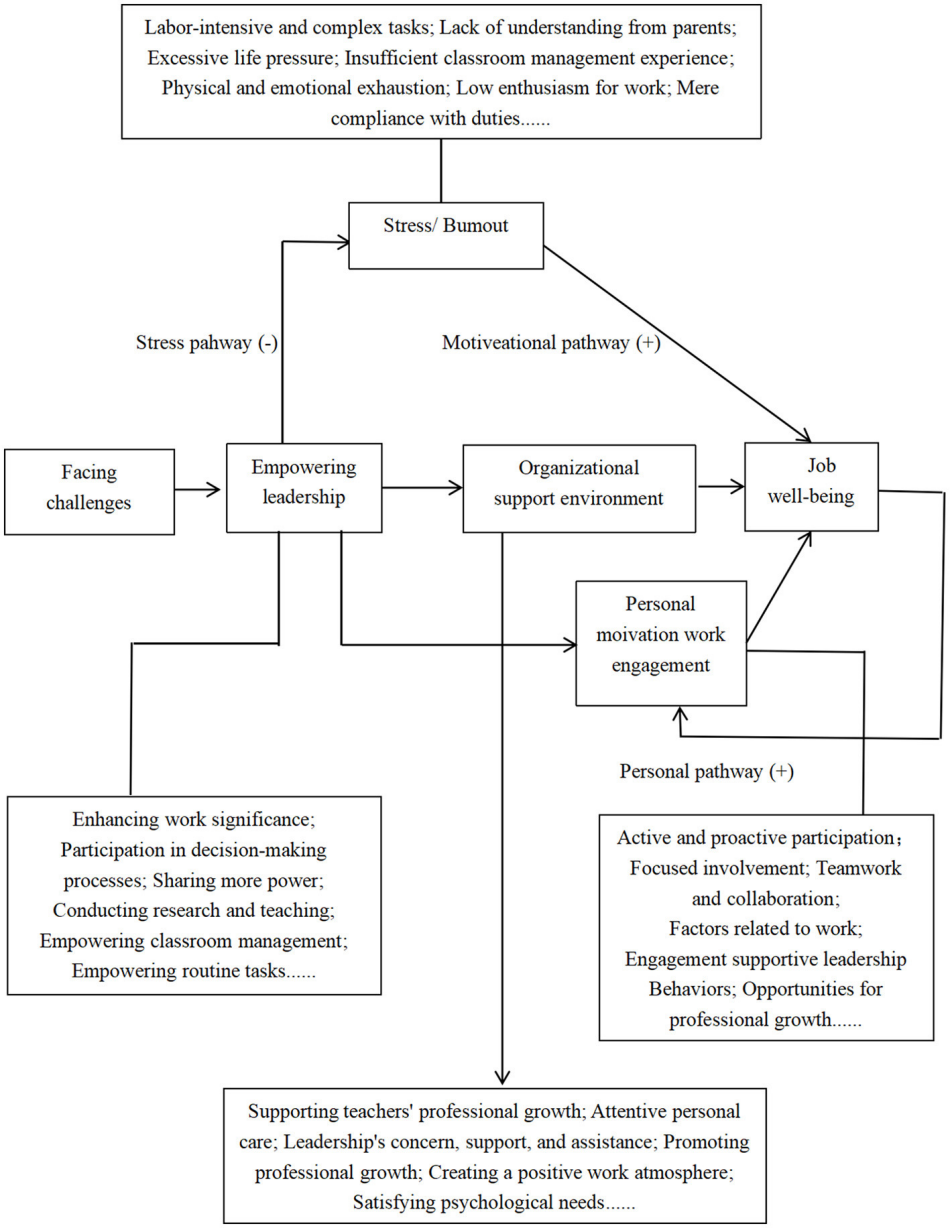
Research model. As shown in this figure, the grounded theory model of preschool teachers' wellbeing illustrates the dynamic relationships between empowering leadership behaviors, job demands, resources, and individual factors. The model underscores how these interrelated elements impact preschool teachers' wellbeing, highlighting both the positive and negative pathways through which leadership behaviors influence job satisfaction, stress, and motivation.

Additionally, in response to RQ3: What factors are related to the wellbeing of kindergarten teachers in China? Regarding the factors related to preschool teachers' wellbeing, we analyzed interview texts and codes using the grounded theory method and integrated the core category of “job wellbeing” chosen by kindergarten principals and preschool teachers. The “storyline” around the core category related to preschool teachers' “job wellbeing” can be summarized as follows: Preschool teachers in kindergartens often face challenges in various aspects, and the empowering leadership behaviors of kindergarten leaders, such as principals, primarily originate from their “leadership behaviors.” These behaviors correlate with the preschool teachers' job wellbeing either positively or negatively through stress pathways, motivational pathways, and individual pathways. Since the factors correlate with the job wellbeing of preschool teachers are individual psychological experiences (24), and the empowering leadership of kindergarten principals involves more empowerment and autonomy, it interacts with the kindergarten organization, environment, and individual factors influencing their behaviors and outcomes. That is, the empowering leadership behaviors of kindergarten leaders may act upon preschool teachers' job wellbeing through factors related to job demands, job resources, and individual characteristics. Therefore, “work stress and burnout,” “organizational support and environment,” and “personal motivation and work engagement” all significantly explain the job wellbeing of preschool teachers (The grounded theory model of preschool teachers' wellbeing is shown in Figure 1).

### 4.1 Bidirectional role of empowering leadership: enhancing and challenging the job wellbeing of preschool teachers

According to the JD-R model theory, leaders should balance employees' job demands and resources in order to foster positive behaviors and outcomes through work-related factors (38). Empowering leadership is a process whereby leaders grant more power to employees through specific leadership behaviors and strive to provide opportunities for autonomous decision-making (39). In this study, we deduced from the interview texts that the factors of empowering leadership correlated with preschool teachers' job wellbeing include “the essence of empowering leadership,” “the content of empowerment,” and “the function and effect of empowerment.” Initially, nine kindergarten principals and teachers stated that empowering leadership is a process that can enhance the work significance for preschool teachers, involve them in decision-making processes, and allow them to experience more power. The results of this study indicate that preschool teachers perceive empowering leadership as a process that enhances their sense of work significance, promoting their appreciation of the existential meaning of their work in kindergartens, and the experience of value and achievement. Furthermore, seven respondents indicated that empowering leadership also facilitates the involvement of preschool teachers in the decision-making processes of kindergarten organizations, allowing them to express their opinions and views on kindergarten work, activities, and tasks (40). However, seven respondents indicated that empowering leadership is a bidirectional process. Some preschool teachers have noted that excessive empowerment can increase their workload and lead to psychological stress (6).

*During this process, I felt quite proud; it gives me a sense of belonging. This empowerment makes me feel accomplished and connected (PR-T10-A1-1)*.

*However, the empowerment from leadership can feel very burdensome. Initially, we agreed that we could do it ourselves, but then after we finished, the plan was suddenly changed multiple times... It's like there's no planning, a lot of it is just impromptu, typical of workplace Pick-up Artist (PU-T1-A13-3)*.

Second, the results of this study revealed that preschool teachers feel empowered by kindergarten principals during the execution of kindergarten research and teaching, routine tasks, autonomous classroom management, and organization of unique kindergarten activities. Six respondents indicated that while conducting teaching activities, the empowering leadership of the kindergarten principals allows them to independently decide on teaching methods, arrangements, and ways to guide child development (41). With the leader's empowerment, four preschool teachers stated that they can develop classroom routines, teaching models, and guidance methods tailored to the developmental characteristics of the children in their classes (42). Moreover, seven preschool teachers indicated that, by granting more empowerment to them, kindergarten leaders stimulate their involvement and authority in designing, conducting, and implementing special thematic activities. The empowerment of early childhood educators promotes the organization of kindergarten activities and enhances their professional skills (43).

*For example, during physical activities or other artistic and language activities, he allows me to assess what is best for our class, or what would be most beneficial for us. He gives me the authority to decide how to organize these activities, which counts as empowerment (PU-T11-A4-1)*.

*Besides pandemic prevention and control, there are many other areas, such as data organization and reporting; these routine tasks can also be delegated. We can arrange them ourselves; after all, the leader does not manage the process, she only cares about the final results (PR-T8-A6-3)*.

Moreover, 14 kindergarten principals and teachers stated that when preschool teachers feel more empowered, it can enhance their work efficiency, foster a team-oriented atmosphere, increase job enthusiasm, and promote professional growth and autonomy. When preschool teachers experience more empowerment, they have greater autonomy in carrying out kindergarten tasks, completing them more efficiently and conveniently. They also feel more empowered and accomplish cooperation and collaboration in an autonomous and democratic atmosphere at the kindergarten (44), which boosts their enthusiasm for work (71). Sixteen kindergarten principals and preschool teachers mentioned that leadership empowerment, combined with understanding children's developmental characteristics, continually updates their knowledge structure and enhances their professional capabilities in early childhood education and teaching (31). If they feel more empowered, they can have more control over teaching activities and classroom management (45), which may make them more proactive, relaxed, and comfortable (46). Here are parts of the verbatim transcripts:

*Empowerment makes me more efficient in my work (PR-T8-A7-3)*.

*For me, empowerment is quite positive because it helps us carry out our work, allows us to perform freely, and I become more proactive in my work (PR-T2-A10-7)*.

*This leader's delegation also contributes to my professional growth, and it helps develop my professional skills, teacher leadership, writing skills, and coordination abilities (PU-T9-A11-3)*.

*For example, in organizing activities or being empowered, there is greater autonomy. Once the task is assigned to me, he does not interfere much, allowing me to freely develop activities based on my own ideas (PR-T5-A12-7)*.

### 4.2 Stress pathway: exploring the interactive relationship between work stress and burnout in preschool teachers' job wellbeing

According to the JD-R model theory, work stress and burnout are job demands faced by preschool teachers in their daily tasks at kindergartens (47). Initially, 15 kindergarten principals and preschool teachers reported that insufficient support from leaders leads to a series of anxious and tense states due to challenging workloads, difficulties in communicating with parents, intense competition in interpersonal relationships, and overwhelming life pressures (48). Such high levels of work stress result in several negative outcomes, including low work enthusiasm, detrimental effects on child development, interpersonal conflicts, difficulties balancing family life, negative personal emotions, and adverse effects on physical health (49). Interviews with preschool teachers showed that providing them with more leadership care and support, sharing emotional experiences with friends and colleagues, engaging in leisure activities, and adjusting personal attitudes can alleviate their stress and tension to some extent (50). Respondents in this study made the following comments:

*When I'm under stress, I find that I'm not fully dedicated during the guidance process; I'm not as focused on the children's development, which can have a negative impact (PU-T7-A23-4)*.

*When I feel a lot of mental pressure, I often experience some depressive emotions, and these feelings tend to build up while I'm working (PU-T7-A26-10)*.

*If it's after work hours, I usually play on my phone or play a round of King of Glory—just play a game to vent. It's a way to relax and relieve the stress (PR-T6-A30-5)*.

*When I'm feeling stressed, I do things I enjoy, even small things that I feel can alleviate that pressure, like spending time with the children (PR-T5-A31-5)*.

When feeling a lack of empowerment, 17 kindergarten principals and preschool teachers stated that they may exhibit signs of physical and emotional exhaustion, low work enthusiasm, and burnout behavior characterized by merely going through the motions (31). Such burnout is often caused by factors such as excessively hard and busy work, long working hours, lack of classroom management experience, absence of professional guidance, confusion about professional growth, lack of recognition and appreciation, and insufficient understanding from parents (51, 52). This type of burnout can lead to severe negative outcomes, including low work efficiency, overwhelming fatigue, identity crises as teachers, negative emotions, and a lack of motivation (53).

*I want to retire now, I'm too tired. This industry is too competitive, and I'm physically and mentally exhausted, I can't take it anymore (PR-T12-A32-3)*.

*It's just that things are too complicated, and then getting up too early in the morning, not getting enough rest, over time, it leads to burnout (PR-T2-A35-3)*.

*Some parents are either uncooperative or just don't understand, which makes me very unhappy. This kind of work brings some negative emotions. Basically, I feel unmotivated, and then I can't seem to find any interest in my tasks (PU-T6-A40-4)*.

### 4.3 Motivational pathway: exploring the interactive relationship between a supportive environment and preschool teachers' job wellbeing

According to the JD-R model, work resources are considered “positive factors” in the workplace, which are elements within the organization that facilitate the achievement of work goals, reduce job demands, and promote employee development, such as organizational support and work atmosphere (47). From the interview transcripts, factors influencing preschool teachers' job burnout were identified, including “experiences of organizational support” and “strategies of organizational support.” Initially, when preschool teachers experience more supportive leadership behaviors, a harmonious work atmosphere, attentive personal care, and concern and support from colleagues, they tend to work harder and conduct their kindergarten duties more autonomously (54). Twenty-three kindergarten principals and preschool teachers noted that more concern, support, and help from leaders, higher management efficiency, more support from colleagues, provision of a good working environment, and promotion of professional growth can enhance their sense of organizational support (55).

*I hope that the leadership can empower me more. When I am responsible for a task, I also hope that the leader can support me, cooperate with my work, and offer more affirmation (PR-T8-A50-5)*.

*I hope to receive more support for my work, especially in terms of the arrangements made in the kindergarten, I hope to get some support from my colleagues (PU-T3-A52-3)*.

### 4.4 Personal pathways: exploring the interwoven relationship between motivation and work engagement in enhancing preschool teachers' job wellbeing

This study drew on the JD-R model theory (47), and through systematic analysis using grounded theory, it was found that personal factors such as individual motivation and work engagement played a positive role between empowering leadership and preschool teachers' job wellbeing. Furthermore, 17 respondents stated that the empowering leadership of the principals may continuously relate to the preschool teachers' personal motivation and work engagement, helping them maintain a positive and proactive experience of job wellbeing (71). Four teachers stated that when preschool teachers perceive more empowering leadership, it enhances the sense of significance in their work and, through participation in decision-making, provides a greater sense of autonomy. This further satisfies their psychological needs, guides them to maintain a positive personal mindset, and motivates them to invest more actively in the educational activities of the kindergarten (56, 57).

*The principal encourages us to make decisions based on our own judgment and style and to carry them out independently. This autonomy not only boosts my motivation but also makes me feel that my work is more meaningful and valuable (PU-T9-A3-1)*.

*In the shadow play activities, the principal chose to empower us, allowing us to independently decide on the specific presentation of the activity. This not only gives us the opportunity to better exercise and showcase our abilities, but also greatly enhances my engagement and involvement (PR-T8-A7-6)*.

## 5 Discussion

### 5.1 Comprehensive discussion

#### 5.1.1 Relationship of stress path factors to preschool teachers' job wellbeing

Based on the JD-R model, this study identified “work stress-work burnout” as a key stress pathway relating to preschool teachers' job wellbeing. The findings indicate that empowering leadership can improve preschool teachers' wellbeing by reducing work stress and burnout. This validates the JD-R model and aligns with previous research (27, 31, 49). For example, the JD-R model is often used in organizational research to explain the relationships between job demands, work resources, and employee wellbeing (47, 58). According to the JD-R model, low-quality leadership can increase job demands but have a negative relationship with organizational outcomes (59). Work stress, regarded as job demands, refers to the negative physiological or psychological responses triggered by organizational demands (33). Therefore, work stress and burnout are key variables correlated with preschool teachers' wellbeing. In China, preschool teachers face high power distance, often receiving directives from superiors, managing pandemic control, and handling parental guidance, all of which lead to increased work stress (49). Empowering leadership helps reduce this power distance, providing teachers with greater autonomy and vitality, which in turn alleviates work stress and burnout, ultimately improving job satisfaction (31). Moreover, during the COVID-19 pandemic, empowering teachers was found to reduce their stress and burnout, thereby enhancing their wellbeing (21, 77). Thus, work stress and burnout are significant stress pathways through which empowering leadership is related to preschool teachers' job wellbeing (71).

#### 5.1.2 Relationship of motivational path factors with preschool teachers' job wellbeing

Through qualitative research, this study identified motivational path factors through which empowering leadership relates to preschool teachers' job wellbeing in China, primarily including organizational support and work environment factors. In the motivational pathways affecting wellbeing, when preschool teachers perceive empowering leadership from kindergarten principals and receive support for professional growth and attentive care for their personal needs, these organizational support experiences—combined with a positive work environment, leadership care, professional development, and supportive work atmosphere—can enhance teachers' proactive engagement and collaboration with colleagues, ultimately improving their wellbeing. In other words, empowering leadership can enhance preschool teachers' job wellbeing by providing organizational support and fostering a positive work environment. This finding is consistent with previous studies (25, 51, 60, 61). According to the JD-R model, high-quality leadership can enrich employees' work resources and has a positive relationship with organizational outcomes (59). Hobfoll (69) also emphasized that perceived organizational support is a key work resource, especially in the education sector, where effective leadership can enrich resources to help teachers better cope with complex and changing situations, thereby improving their service quality and performance. Preschool teachers face higher demands in terms of energy, emotions, and professionalism, and are often under dual pressure from work demands and early childhood education. Given that their work demands cannot be easily reduced, empowering leadership is more effective in terms of granting them autonomy, enhancing their vitality and sense of support, and fostering a positive work environment to help them face current challenges with enthusiasm (31). In the face of industry challenges and changes, perceived organizational support can contribute to improving job wellbeing (57). Therefore, empowering leadership can influence preschool teachers' job wellbeing through the motivational pathways of organizational support and work environment.

#### 5.1.3 Relationship of personal path factors on preschool teachers' job wellbeing

Through qualitative research, this study identified personal path factors through which empowering leadership is related to the job wellbeing of preschool teachers in China, mainly including personal motivation and work engagement. For example, in the personal pathways affecting job wellbeing, when preschool teachers perceive empowering leadership from kindergarten principals, it can stimulate their enthusiasm and intrinsic motivation, leading to greater engagement in their work and ultimately improving their wellbeing. In other words, empowering leadership can enhance preschool teachers' job wellbeing through personal motivation and work engagement, consistent with previous studies (62–64). According to Schaufeli (59), high-quality leadership can increase employees' work resources, thereby boosting their motivation. This study confirms that empowering leadership is related to preschool teachers' wellbeing through personal factors such as work engagement, similar to previous research findings. For instance, Radic et al. (58) found that personal factors, such as work engagement, contribute to greater job wellbeing; employees who are more focused and engaged in their work often report higher wellbeing. Similarly, Alotaibi et al. (67) found that employees who perceive higher levels of empowering leadership are more likely to invest their energy, focus, and enthusiasm in their work, contributing positively to the organization. Therefore, when preschool teachers perceive high levels of empowering leadership, it can boost their motivation and work engagement, ultimately enhancing their wellbeing (65). Nong et al. (72) noted that providing supportive work environments and conditions for preschool teachers, along with more care, helps improve their motivation, resulting in more positive emotions and greater enthusiasm for their work, thereby promoting higher levels of wellbeing. In other words, a higher level of empowering leadership can correlate with preschool teachers' job wellbeing by enhancing their personal work engagement (71). Therefore, empowering leadership can correlate with preschool teachers' job wellbeing through personal factors such as motivation and work engagement.

### 5.2 Implications and contributions

This study fills a significant gap in the literature on the JD-R model by exploring the relationships between organizational support, work engagement, job wellbeing, and personal motivation/work engagement among preschool teachers in the kindergarten context. While previous studies on the JD-R model primarily focused on work demands and resources, this study incorporated personal motivation and work engagement into the JD-R framework, providing a deeper understanding of how these factors correlate with job wellbeing within early childhood education. Second, this study extends the JD-R theoretical model by further exploring the motivational role of organizational support and the work environment. We found that empowering leadership enhances organizational support, which in turn has a positive relationship with preschool teachers' work engagement and job wellbeing. This finding aligns with prior research showing that effective leadership and work environment contribute significantly to employee motivation and wellbeing. By focusing on early childhood education, this study highlights the important role of work resources—specifically, organizational support—in mitigating the high demands placed on preschool teachers. Third, this study contributes to the literature by examining the bidirectional effects of personal resources on individual behavioral outcomes within the JD-R model. While previous studies largely focused on how personal resources influence positive behaviors and outcomes, this study adds to the understanding of how empowering leadership can leverage personal resources such as motivation and work engagement to enhance job wellbeing (62, 63). The findings suggest that empowering leadership has a positive relationship with preschool teachers' wellbeing by enhancing their personal motivation and engagement, supporting the argument that leadership plays an integral role in activating personal resources for positive outcomes.

From the perspective of organizational psychology, this study emphasizes that leadership is a key factor in coping with crises and improving wellbeing. Based on our qualitative findings, kindergarten leaders can enhance the positive effects of empowering leadership by focusing on stress, motivational, and personal pathways. Empowering leadership can help reduce work stress, enhance motivation, and foster personal resources, ultimately improving job wellbeing for preschool teachers. This supports the argument that targeted leadership strategies are essential for enhancing the wellbeing of early childhood educators. However, it is also essential for kindergarten principals to balance empowerment and avoid over-delegation, as excessive authority or autonomy may increase role ambiguity and stress among preschool teachers. Principals should actively leverage the positive outcomes of balanced empowering leadership to improve both organizational performance and teacher wellbeing. By providing more autonomy, along with adequate guidance and support, leaders can foster a sense of purpose and commitment among preschool teachers, which has been shown to enhance job satisfaction and reduce burnout. Furthermore, educational managers should focus on creating a supportive work environment that enhances teachers' professional growth, improves working conditions, and provides resources for emotional management. Specifically, kindergarten principals should aim to provide professional development opportunities, clear goal orientation, and sufficient emotional support. By doing so, they can reduce work-related stress and promote a healthier work environment, which is key to improving preschool teachers' wellbeing (31, 57). To enhance preschool teachers' job wellbeing, empowering leadership should also aim to provide sufficient organizational resources. These resources can include strategies for managing stress, opportunities for career advancement, and measures to improve the working environment. Providing these resources will not only improve wellbeing, but also motivate preschool teachers to actively engage with their work, ultimately benefiting the quality of early childhood education.

In conclusion, this study provides practical insights for educational administrators and policymakers. By focusing on empowering leadership, organizational support, and the provision of work resources, kindergarten principals can enhance preschool teachers' job wellbeing. This approach is particularly important in China, where preschool teachers face high levels of work stress due to both caregiving and educational responsibilities. Effective leadership that balances empowerment with support can reduce stress, foster motivation, and create a positive work environment, ultimately leading to better wellbeing for preschool teachers and improved educational outcomes.

### 5.3 Limitations and future research directions

This study has several inherent limitations that need to be acknowledged. First, the use of a small qualitative sample is a limitation, as it restricts the broader generalizability of the findings. While the sample size was sufficient to achieve theoretical saturation in grounded theory research, the findings may not be fully representative of all preschool teachers in China. The grounded theory approach provided in-depth insights into the factors related to preschool teachers' job wellbeing; however, the small sample size may limit the conclusions to specific contexts. Future research could address this limitation by using larger quantitative samples or employing mixed-method approaches to validate and expand on these findings, thus providing additional insights and uncovering region-specific differences and more varied experiences across different types of educational settings. Second, the reliance on qualitative interviews may have introduced subjective biases, as participants' responses could be influenced by their personal experiences or perceptions of the research context. Future studies could use longitudinal data collection to track changes in teachers' wellbeing over time, which would provide a more dynamic understanding of how job wellbeing evolves and how different leadership styles are related to this evolution (71). Furthermore, from the perspective of positive psychology, providing a good work environment and conditions can not only ignite teachers' enthusiasm and proactivity, but can also enhance their work performance. Future studies could investigate how specific work environment improvements or support measures could further enhance preschool teachers' job wellbeing, particularly in the face of challenges such as workload and resource constraints. By focusing on practical interventions that improve the work environment (66), future research can contribute to developing actionable strategies that educational managers can implement to improve teachers' wellbeing and job satisfaction (15).

## 6 Conclusion

In this study, we conducted semi-structured interviews with 12 kindergarten principals and 12 preschool teachers, using grounded theory qualitative research analysis methods to explore the factors related to preschool teachers' job wellbeing. Therefore, the qualitative research component utilized grounded theory systematic analysis and coding integration, focusing on preschool teachers' job wellbeing as the storyline. The findings suggest that the empowering leadership of kindergarten principals might be related to preschool teachers' job wellbeing through stress pathways such as work pressure and burnout. It might also affect the preschool teachers' stress factors, individual factors, and job wellbeing through motivational pathways such as enhancing organizational support and work environment. The empowering leadership of kindergarten principals could also be related to preschool teachers' job wellbeing through personal factors such as personal motivation and work engagement. The relationship between principals' empowering leadership and preschool teachers' job wellbeing may also have a continuous relationship with their personal motivation and work engagement, maintaining a positive and proactive job wellbeing experience.

## Data Availability

The raw data supporting the conclusions of this article will be made available by the authors, without undue reservation.
