# Supplementary material for: Constructing a model of the factors related to the job wellbeing of preschool teachers in China: a grounded theory study
Source: Front Public Health. 2025 Jan 15;12:1497629. doi: 10.3389/fpubh.2024.1497629 (PMC11774746; doi:10.3389/fpubh.2024.1497629)
Supplement: Supplementary file 1 [file Table_1.docx]

**Appendix 1 Data Coding**

| **Main Category** | **Subcategory** | **Category** | | **Frequency** |
| --- | --- | --- | --- | --- |
| Empowering Leadership | Essence of Empowerment | Enhancing work significance | | 9 |
|  |  | Participation in decision-making processes | | 7 |
|  |  | Sharing more power | | 12 |
|  | Content of Empowerment | Conducting research and teaching | | 6 |
|  |  | Empowering classroom management | | 4 |
|  |  | Empowering routine tasks | | 7 |
|  | Relationship between empowerment and well-being | Effective decentralized management | | 7 |
|  |  | Improving job performance | | 8 |
|  |  | Creating a positive work atmosphere | | 6 |
|  |  | Fostering professional growth | | 10 |
|  |  | Enhancing work enthusiasm | | 6 |
| Work Stress | Sources and Dilemmas of Work Stress | Labor-intensive and complex tasks | | 5 |
|  |  | Lack of understanding from parents | | 5 |
|  |  | Excessive life pressure | | 5 |
|  |  | Insufficient classroom management experience | | 4 |
|  |  | Lack of personal knowledge | | 5 |
|  | Relationship between work stress and well-being and Challenges of Work Stress | Low work enthusiasm | | 5 |
|  |  | Negative effect on child development | | 4 |
|  |  | Insufficient parental guidance | | 4 |
|  |  | Negative personal emotions | | 6 |
|  |  | Resignation and career changes | | 3 |
| Job Burnout | Characteristics of Job Burnout | Physical and emotional exhaustion | | 4 |
|  |  | Low enthusiasm for work | | 6 |
|  |  | Mere compliance with duties | | 7 |
|  | Strategies for Addressing Job Burnout | Improving the work environment | | 5 |
|  |  | Satisfying psychological needs | | 10 |
|  |  | Promoting professional growth | | 7 |
| Organizational Support | Experiences of Organizational Support | Supporting teachers' professional growth | 9 | |
|  |  | Attentive personal care | 4 | |
|  |  | Leadership's concern, support, and assistance | 7 | |
|  | Strategies of Organizational Support | Promoting professional growth | 8 | |
|  |  | Creating a positive work atmosphere | 4 | |
|  |  | Satisfying psychological needs | 4 | |
| Work Engagement | Experiences of Work Engagement | Active and proactive participation | 11 | |
|  |  | Focused involvement | 6 | |
|  |  | Teamwork and collaboration | 4 | |
|  | Factors related to Work Engagement | Supportive leadership behaviors | 6 | |
|  |  | Opportunities for professional growth | 5 | |
|  |  | Harmonious organizational atmosphere | 9 | |
|  |  | Insufficient compensation | 5 | |
| Factors related to Job Well-Being | Factors related to Job Well-Being | Increased empowerment and management | 9 | |
|  |  | Positive work environment | 7 | |
|  |  | Harmonious interpersonal relationships | 7 | |
|  |  | Opportunities for professional growth | 5 | |
|  |  | Recognition and support from society | 6 | |
|  |  | Support from family | 4 | |
|  |  | Positive personal attitude | 7 | |
|  |  | Inadequate salary and benefits | 10 | |
|  |  | Excessive workload | 10 | |
